# The Usability and Effectiveness of Mobile Health Technology–Based Lifestyle and Medical Intervention Apps Supporting Health Care During Pregnancy: Systematic Review

**DOI:** 10.2196/mhealth.8834

**Published:** 2018-04-24

**Authors:** Sanne B Overdijkink, Adeline V Velu, Ageeth N Rosman, Monique DM van Beukering, Marjolein Kok, Regine PM Steegers-Theunissen

**Affiliations:** ^1^ Department of Obstetrics and Gynecology Erasmus Medical Center Rotterdam Netherlands; ^2^ Academic Medical Center Department of Obstetrics and Gynecology University of Amsterdam Amsterdam Netherlands; ^3^ Division of Neonatology Department of Pediatrics Erasmus Medical Center Rotterdam Netherlands

**Keywords:** mHealth, pregnancy, lifestyle, health care, maternal health

## Abstract

**Background:**

A growing number of mobile health (mHealth) technology–based apps are being developed for personal lifestyle and medical health care support, of which several apps are related to pregnancy. Evidence on usability and effectiveness is limited but crucial for successful implementation.

**Objective:**

This study aimed to evaluate the usability, that is, feasibility and acceptability, as well as effectiveness of mHealth lifestyle and medical apps to support health care during pregnancy in high-income countries. Feasibility was defined as the actual use, interest, intention, and continued use; perceived suitability; and ability of users to carry out the activities of the app. Acceptability was assessed by user satisfaction, appreciation, and the recommendation of the app to others.

**Methods:**

We performed a systematic review searching the following electronic databases for studies on mHealth technology–based apps in maternal health care in developed countries: EMBASE, MEDLINE Epub (Ovid), Cochrane Library, Web of Science, and Google Scholar. All included studies were scored on quality, using the ErasmusAGE Quality Score or the consolidated criteria for reporting qualitative research. Main outcome measures were usability and effectiveness of mHealth lifestyle and medical health care support apps related to pregnancy. All studies were screened by 2 reviewers individually, and the guidelines of the Preferred Reporting Items for Systematic Reviews and Meta-Analyses statement were followed.

**Results:**

Our search identified 4204 titles and abstracts, of which 2487 original studies remained after removing duplicates. We performed full-text screening of 217 studies, of which 29 were included in our study. In total, 19 out of 29 studies reported on mHealth apps to adopt healthy lifestyles and 10 out of 29 studies to support medical care. The lifestyle apps evaluated in 19 studies reported on usability and effectiveness: 10 studies reported positive on acceptability, and 14 studies reported on feasibility with positive results except one study. In total, 4 out of 19 studies evaluating effectiveness showed significant results on weight gain restriction during pregnancy, intake of vegetables and fruits, and smoking cessation. The 10 studies on medical mHealth apps involved asthma care, diabetic treatment, and encouraging vaccination. Only one study on diabetic treatment reported on acceptability with a positive user satisfaction. In total, 9 out of 10 studies reported on effectiveness. Moreover, the power of most studies was inadequate to show significant effects.

**Conclusions:**

Most studies on mHealth apps to support lifestyle and medical care for high-income countries reveal the usability of these apps to reduce gestational weight gain, increase intakes of vegetables and fruit, to quit smoking cessation, and to support health care for prevention of asthma and infections during pregnancy. In general, the evidence on effectiveness of these apps is limited and needs further investigation before implementation in medical health care.

## Introduction

### Mobile Health in Developed Countries

Mobile health (mHealth) technology–based apps are becoming rapidly available, especially in high-income countries. mHealth was defined by the World Health Organization as the use of mobile devices (mobile phones, patient monitoring devices, and personal digital assistants) for medical and public health practice [1]. Most of the mHealth apps aim to adopt healthy lifestyles such as nutrition, weight control, and smoking cessation, or to support medical health care such as the control of glucose levels to support diabetic care [2,3]. The benefits of mHealth apps include that they can be delivered to an individual anywhere at any time and provide opportunities for interaction and tailoring of specific domains and target groups. Several mHealth apps have been developed related to pregnancy and as such have the potential to improve maternal health care [4].

The use of mobile phones is increasing worldwide [5]. It is estimated that in 2020, 90% of the worldwide population will own a mobile phone [5]. In 2015, about 94% of the Dutch population aged between 25 and 45 years owned a smartphone with internet access offering opportunities for a broad use of mobile apps including health apps [6]. Carroll et al showed that main users of health apps were healthy, young, higher-educated persons with a higher income. However, they also showed that in general, determinants such as gender, age, and education were less suitable for predicting the use of mobile and health apps, which is in contradiction with the profile of main users of health apps [5].

### Mobile Health During Pregnancy

There are more apps available to support pregnancy than for any other medical domain [7]. Apps can contribute to healthy lifestyle during pregnancy, as pregnancy is a critical teachable period in the lives of young women [8]. App use is often associated with intentions to change diet and physical activity. However, the quality, reliability, and effectiveness of current available pregnancy apps are undetermined. Therefore, exposure to potential harmful apps or participation in research with nonevidence-based mobile apps should be carefully considered, especially during pregnancy when women are more sensitive for external influences [9]. Moreover, unnecessary information and advice on lifestyle and health care can lead to more worrying and stress during pregnancy. Therefore, information on usability and effectiveness is crucial for implementation of new apps in maternal health care. This was underlined by an evaluation of 10 popular, free maternal and baby-health apps by Scott et al [9]. A health professional was involved in the development of only 4 apps, and the content was evidence-based in 3 apps. Bert et al found that only less than half of the reviewed apps presented a privacy policy statement, whereas a scientific board was mentioned in a third of the apps [8].

From this background, we conducted a systematic review to provide evidence on the usability, that is, feasibility and acceptability, and effectiveness of mHealth lifestyle and medical apps to support health care during pregnancy in high-income countries. We used the United Nations Country classification to establish which countries are considered high income [10].

## Methods

The review protocol was registered on PROSPERO (registration number CRD42016053325). The authors followed the guidelines for Preferred Reporting Items for Systematic Reviews and Meta-Analyses statement [11].

### Search Method

We conducted a systematic review of studies on mobile lifestyle and medical apps to support health care during pregnancy in developed countries. In our study, all text messaging services, intervention, or monitoring system with the intention to improve maternal health during pregnancy were considered apps. We searched the databases EMBASE (1947-2017), MEDLINE Epub (Ovid) (1946-2017), Cochrane Library (1992-2017), Web of Science (1900-2017), and Google Scholar, using a combination of Medical Subject Headings topics and free text terms. The literature search was performed in February 2016 according to a predefined protocol with the aid of a librarian of the Erasmus MC, the Netherlands, and updated in February 2017. A copy of the complete search strategy including search terms is available in Multimedia Appendix 1. The search was limited to human studies, reported in the English language, and no time restrictions were applied.

### Inclusion and Exclusion Criteria

We included original research and qualitative health care research studies on mHealth technology–based apps for pregnant women with the aim to support lifestyle and health care during pregnancy. The inclusion criteria were (1) pregnant women and partner; (2) an app or text message service during pregnancy; (3) studies that were randomized (controlled) trials, pilot studies, prospective or retrospective cohort studies, surveys, and qualitative health care research; (4) original research; and (5) outcomes include information on feasibility, acceptability, and the effectiveness of mHealth apps.

The exclusion criteria were as follows: (1) apps for professionals; (2) studies in developing countries; (3) apps or text message service starting before or after pregnancy; (4) use of mobile phone only for contacting health care providers; and (5) review articles, editorials, letters, comments, and textbook articles.

### Study Selection

To evaluate the feasibility and acceptability, we established definitions for these terms to be able to uniformly compare the evidence of the included studies. These definitions were based on the study by Bowen et al in 2010 and adjusted for our research on apps [12]. Feasibility was defined as actual use, interest, intention, and continued use, perceived appropriateness, and ability of users to carry out the activities of the app. Acceptability was assessed by user satisfaction, appreciation, and the recommendation of the app to others.

### Data Extraction and Quality Assessment

Studies were selected in a 2-stage process. First, 2 reviewers (AV and SO) independently screened the titles and abstracts for relevance to our criteria. Hence, the full manuscripts were studied by the same reviewers. In case full text was not available, the corresponding author was contacted to request for the article. Discrepancies were resolved by a third senior reviewer (AR). We completed our search by checking the references of the included articles for studies not found in the search of the included articles. For data extraction, a standardized form, adjusted for this particular study, was used. Data extraction was performed by both reviewers individually. Differences were resolved by consensus.

The ErasmusAGE quality assessment tool for systematic reviews was used to assess the quality of the intervention and observational studies. This quality score for systematic reviews is enclosed in the Multimedia Appendix 2. The ErasmusAGE quality score is composed of 5 items. Each item is allocated 0, 1, or 2 points [13-15]. This summarizes a total score between 0 and 10 points in which 10 points represent the highest quality. For the qualitative research articles, the consolidated criteria for reporting qualitative research (COREQ) was used to evaluate the quality [16].

## Results

### Study Selection

**Figure 1:**
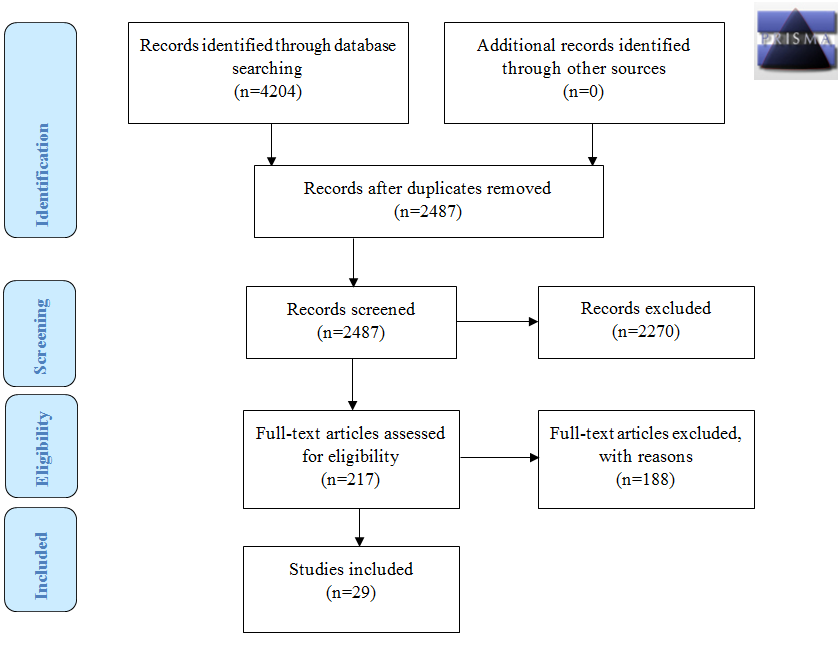
Preferred Reporting Items for Systematic Reviews and Meta-Analyses (PRISMA) flow diagram.

We identified 4204 titles and abstracts of which 2487 original articles remained after removing duplicates (Figure 1). In total, 217 of the articles were assessed for eligibility by full-text screening. The full-length articles were assessed by both reviewers. Some studies used the same app within an identical study population. In this case, only the most recent and/or most complete study was selected for data extraction. After full-text screening, 28 articles were included. There was some doubt about 1 full-text article, which was therefore presented to the third reviewer. The study had a different design but did focus on a wide array of lifestyle factors. Patients could use mobile phones to text pregnancy-related questions to a programmed system after which the patient received either a direct answer or encouragement to seek answers from health care providers. The third reviewer concluded that the design of the study (texting questions to a programmed system and reporting a follow-up of the effectiveness of the text messages) conformed to the inclusion criteria and therefore the study was included.

This resulted in 29 included articles for further evaluation. Manual searching of the reference list of the included papers did not yield additional papers for inclusion in our analysis.

### Data Extraction

All original papers were analyzed on the 2 outcome measures usability, that is, feasibility and acceptability, and effectiveness of mHealth lifestyle apps and medical apps related to pregnancy. In total, 19 studies reported on the usability and effectiveness of mHealth lifestyle and 10 studies on medical apps. The study characteristics of the studies reported on mHealth lifestyle apps are presented in Multimedia Appendix 3, and the study characteristics of the studies reported on mHealth medical apps are reported in Multimedia Appendix 4. We report first the results on feasibility, followed by acceptability and effectiveness.

### Principal Findings of the Studies on Usability and Effectiveness of mHealth Lifestyle Apps

A complete overview of the results on mHealth lifestyle apps can be found in Multimedia Appendix 5. This appendix provides detailed information such as country, sample size, study design, study setting, etc.

#### Results on Feasibility of mHealth Lifestyle Apps

##### Actual Use

In total, 9 studies reporting on lifestyle behaviors paid attention to the actual use of the mHealth app. These studies focused on smoking cessation [17-25], nutrition [17,18], weight control [23], and physical activity [20]. All studies reported that the study participants opened and responded to the messages they received; however, responding to the messages varied from 59 to 100% of all participants in the study.

##### Interest or Intention to Use or (Intent) Continued Use

In total, 8 studies reported on interest or intention to use of the app offered by the mHealth app [19,20,22,24,26-29]. These studies focused on nutrition [19,26], vitamin use [26], smoking cessation [22,24,29], to stop alcohol consumption [19,26], physical activity [20], weight control [27,28], and mental health [24]. Participants agreed that they were interested in the service and that they planned to continue being enrolled; however, the study by Choi et al showed that the response rate dropped to 24% after 10 weeks of participation. This study focused on stimulating physical activity by sending daily messages.

##### (Perceived) Suitability

In total, 4 studies reported on suitability [22,25,29,30]. In total, 3 studies focused on smoking cessation by sending informative text messages [22,25,29]. The study by Song et al had a different design that focused on a wide array of lifestyle factors, that is, vitamin intake, morning sickness, nutrition, and abdominal pains [30]. Patients could use mobile phones to text pregnancy-related questions to a programmed system after which the patient received either a direct answer or encouragement to seek answers from health care providers.

Regarding the smoking cessation studies, 24% of the participants in the MiQuit study thought that the texts were annoying and 26% felt they had received too many messages [22]. Most participants (88%) of the study by Abroms et al judged the number of received messages “just right” [25]. The second study by Naughton et al reported that the approach of participants by the app was appropriate [29].

##### Ability of Participants to Carry out Intervention Activities

In total, 6 studies reported on the ability to use the mHealth app. These studies concentrated on nutrition, folic acid supplement use, to stop alcohol consumption [17,19,30], physical activity [19], weight control [27], smoking [24,29], and mental health [24]. Most participants agreed that the app was simple to use, easy to understand, or user-friendly (Multimedia Appendix 6).

#### Results on Acceptability of mHealth Lifestyle Apps

##### User Satisfaction

In total, 8 studies reported on users’ satisfaction. For smoking cessation [22-26] as well as for nutrition [26] and weight control [21,27,31], the satisfaction of the participants was high. Participants described the app as helpful or useful. In the study by Herring et al [31], participants reported that the intervention was extremely successful in changing eating habits.

##### Suitability

In total, 7 studies reported on (perceived) suitability. These studies focused on smoking cessation [17,23,25,26], nutrition [17,26], vitamin supplement use [17,26], to stop alcoholic consumption [17,26], and weight control [27,28]. Suitability of the app was described by participants as acceptable, liked, reliable, enjoyed the app, and very or somewhat interesting. The study by Bot et al showed a 78% rate of high or intermediate appropriation [18]. The intervention in the study by Soltani et al was liked because of the holistic nature of the program [28]. Within this study, participants received daily text messages supported with appointments with healthy lifestyle midwives, diet and activity goal setting, and self-monitoring diaries.

##### Recommendation to Others

In total, 3 studies reported whether participants would recommend the app to others [21,23,25]. The study by Pollak et al, which focused on weight control by short message service (SMS)-texting interventions, was recommended by 80% of the participants to others [21]. An earlier study by Pollak et al reached a recommendation rate of 78% by all participants. In this study, SMS-delivered support messages were compared with support messages plus a scheduled gradual reduction of smoking. In both arms of the study, the recommendation of the intervention was high [23].

Multimedia Appendix 7 gives an overview of the results of the review on acceptability of mHealth lifestyle apps by summarizing the results per first author, year of publication, used technique of the app, focus of the reported study, and acceptability by study participants, defined as user satisfaction, suitability, and recommendation.

#### Results on Effectiveness of mHealth Lifestyle Apps

In total, 10 studies reported on effectiveness of the app [20-23,28,31-35]. Moreover, 5 studies reported on smoking cessation [22,23,32,33,35]. Naughton et al could not show significant differences, and Pollak et al could not do any statistical analysis due to small groups but showed a lower prevalence of smoking cessation in the intervention group [22,23]. Women enrolled in the study by Moniz et al received 12 weekly text messages encouraging preventive health behaviors. An improvement in self-reported health behaviors was observed between baseline and follow-up, including decreased tobacco use, more prenatal vitamin intake, and more frequent healthy food intake [33].

Fuijoka et al showed significant decreases of carbon monoxide exhalation levels of the participants within 3 months of participation in the study [32].

In total, 3 studies reported on controlled gestational weight gain during pregnancy [21,28,31]. Pollak et al showed a nonsignificant difference in the mean gestational weight gain of 6 pounds less for women who completed the intervention [21]. Soltani et al could not do any statistical analysis due to a small sample size, but the mean gestational weight gain in the intervention group was 6.65 kg vs 9.74 in the control group [28]. Herring et al showed significant differences in gestational weight gain in the intervention group vs the control group (*P*=.03) [31].

Physical activity and nutrition were reported in 2 studies [20,35]. Choi et al compared the use of Fitbit only vs an app plus Fitbit in a group of pregnant women. The intervention group tended to increase in daily steps compared with the Fitbit-only group; however, the difference was not significant [20].

Van Dijk et al reached 1275 couples contemplating pregnancy and 603 pregnant couples. Lifestyle behaviors, ie, folic acid, tobacco and alcohol use, and inadequate nutrition, ie, fruits and vegetables intake, were identified at baseline [35]. After this, a Web-based coaching was created for each user on the most prevalent inadequate nutrition and lifestyle behaviors for 6 months. After 6 months of coaching, intakes increased by 26.3% and 38.4% for vegetable and fruit intake, 56.3% for folic acid supplement use, and 35.1% and 41.9% for reduced tobacco use and alcohol consumption. The program showed the strongest success in women of participating couples.

Evans et al used the TexT4baby program, consisting of 3 text messages per week additional to the regular TexT4baby program, tailored by the date of enrollment and gestational age. They showed only a significantly lower alcohol consumption in the high-dose intervention group, that is, patients receiving the maximum number of messages, but not on other health behaviors, including taking prenatal vitamins, eating 5 or more fruits and vegetables daily, and smoking behavior [34].

In total, 2 studies evaluated the effectiveness of text messages promoting healthy lifestyle behavior in general during pregnancy [33,34].

Multimedia Appendix 8 gives an overview of the results of the review on the effectiveness of mHealth lifestyle apps by summarizing the results per first author, year of publication, used technique of the app, focus of the reported study, and effectiveness based on patient-reported questionnaires. Van Dijk et al, Herring et al, and Fuijoka et al showed significant results on effectiveness of the lifestyle mHealth app. All other studies reported nonsignificant differences [35,31,32].

### Principal Findings of the Studies on mHealth Medical Apps

A complete overview of the results on mHealth medical apps can be found in Multimedia Appendix 9.

#### Results on Feasibility of Medical Apps

Table 1 gives an overview of studies reporting on the feasibility of medical apps by summarizing the results per first author, year of publication, used technique of the app, focus of the reported study, and feasibility reported by patients. Thereafter, we discuss the actual use, interest or intention to use or (intent to) continued use, perceived suitability, and ability of participants to carry out intervention activities of the included studies. In this case, only 1 study reported on the actual use and interest of the app in medical health care [33].

##### Actual Use

Nicholson showed that 65% of the participants logged in to the website at least 3 times during pregnancy. Women with gestational diabetes used the gestational management system (GooDMomS) in which they received Web lessons, self-tracking of weight and glucose, automated feedback, and access to a message board for peer support [36].

##### Interest or Intention to Use or (intent to) Continued Use

Most women in the study by Nicholson did not have experiences with this kind of medical apps, but they were willing to give it a try as participating would not be harmful and maybe others may know more than themselves [36].

**Table 1:** Overview of studies reporting on medical (interventions) apps related to pregnancy: feasibility.

Author and year	Technique	Focus	Feasibility			
			Actual use	Interest	Suitability	Ability
Nicholson et al, 2016 [36]	Web lessons, self-tracking of weight and glucose, automated feedback, and access to a message board for peer support	Diabetes	In total, 65% of the participants logged in to the website at least 3 times during pregnancy	“Using this program would probably…would be the first for me because I don’t do the message boards and things of that nature, but I’m willing to give it a try, just, you know, because somebody may know something more than I do, and it never hurts to ask.”	-	Most participants (*n*=8) thought the website was user-friendly and easy to access

##### (Perceived) Suitability

This outcome was not reported in any of the studies about medical apps.

##### Ability of Participants to Carry out Intervention Activities

Participants in the study by Nicholson et al thought the website was user-friendly and easy to access but logging into the system was sometimes challenging. Access through mobile phone could enhance their ability to login consistently [36].

#### Results on Acceptability of Medical Apps

Table 2 gives an overview of studies reporting on the acceptability of medical apps by summarizing the results per first author, year of publication, used technique of the app, focus of the reported study, and acceptability reported by patients. Thereafter, we discuss the user satisfaction, appreciation, and recommendation of the included studies.

In total, 2 studies reported on acceptability of the medical apps [33,34].

#### User Satisfaction

Hirst et al described an interactive, smartphone-based remote blood glucose monitoring system [37]. Women with gestational diabetes reported their blood glucose levels by telephone on a secure website to a diabetes midwife or physician. This website was checked at least 3 times a week. If required, the midwife or physician contacted the women via SMS or a phone call. In total, 45 out of 49 women agreed their care was satisfactory and the best for them, 47 out of 49 and 43 out of 49 agreed the equipment was convenient and reliable, respectively. Moreover, 42 out of 49 agreed that gestational diabetes mellitus (GDM) health fitted into their lifestyle, and 46 out of 49 agreed that they had a good relationship with their care team [37].

#### Appreciation

In the study by Hirst et al, 83% of the participants agreed or strongly agreed the management system was reliable [37]. In the study by Nicholson et al, women expressed that the intervention materials were useful, well received, and led to a better understanding of how gestational weight gain during pregnancy might affect their child. Logging into the system was sometimes challenging [36].

#### Recommendation

No studies reported recommendations of the medical apps.

### Effectiveness of Medical Apps

One study involved a telehealth program developed to manage asthma in pregnancy. It involved care of respiratory function, supported by a handheld respiratory device and a smartphone app. The primary outcome was change in asthma control as measured by the 7-item Asthma Control Questionnaire-7 at 3 and 6 months. At 6 months, the telehealth program group had significantly better asthma control compared with usual care group (*P*=.02) [38].

In total, 5 studies described an app for pregnant women with diabetes mellitus (type 1 or 2) or GDM [36,39-42]. All studies in this subgroup evaluated an internet-based telemedicine system to monitor and transmit results to a health care professional. In all systems used in this subgroup, a form of personal interaction with a health care professional was possible, mostly by means of text messaging. All 4 studies used blood glucose and/or HbA1C as an outcome measure. Some also assessed insulin use and neonatal outcomes. Carral et al found less insulin use and fewer health care visits in the intervention group; however, there was no significant difference in HbA1C [42]. Homko et al found no significant difference in blood glucose and HbA1C [39]. In contrast to Carral et al, they did find that the proportion of women needing insulin therapy was significantly higher (*P*<.05) in the intervention group. A later study by Homko et al reported no significant differences in glucose values or infant weight [41].

Perez-Ferre et al did not find any differences in HbA1C and blood glucoses or neonatal outcomes [40]. In total, 3 studies evaluated the effectiveness of an app to encourage pregnant women to receive an influenza vaccination during pregnancy [43-45]. In the study by Stockwell et al, a sequence of 5 weekly, automated text messages providing information and reminders about the influenza vaccine were sent [43]. The intervention in the study by Jordan et al used text reminders and tailored education [44]. The study by Yudin et al used 2 weekly sent text messages, during a period of 4 weeks [45].

**Table 2:** Overview of studies reporting on medical (interventions) apps related to pregnancy: acceptability.

Author and year	Technique	Focus	Acceptability	
			User satisfaction	Appreciation
Hirst et al, 2015 [37]	Using a GDM^a^ health system for monitoring all blood glucoses and communication with the research team	Diabetes	In total, 90% of the participants agreed or strongly agreed the management system is convenient	In total, 83% of the participants agreed or strongly agreed the management system is reliable
Nicholson et al, 2016 [36]	Web lessons, self-tracking of weight and glucose, automated feedback, and access to a message board for peer support	Diabetes	-	Women reported little to no experience with online discussion groups, but expressed a willingness to use a message board to communicate with other women with GDM

^a^GDM: gestational diabetes monitoring.

Jordan et al showed an increase of continued intention to be vaccinated as a result of the encouraging messages with an adjusted odds ratio of 2.1 (95% CI 1.4-3.1) but no increased odds of vaccination at follow-up [44]. Stockwell et al reported a higher vaccination-rate, with an adjusted odds ratio of 1.3 (95% CI 1.003-1.69) in favor of the intervention group [43]. Yudin et al did not find significant differences in vaccination rate between the intervention and control group [45] (Table 3).

### Quality of Evidence

Only studies that evaluated a clinical outcome were assessed on quality by the ErasmusAGE quality assessment tool (Table 4). We aimed to evaluate the quality of qualitative research articles by the COREQ. Unfortunately, this was not possible because none of the included studies mentioned reporting according to this guideline [16].

**Table 3:** Overview of studies reporting on medical (interventions) apps related to pregnancy: effectiveness.

Author and year	Technique	Focus	Effectiveness
Zairina et al, 2015 [38]	Telehealth program in which daily lung functions were recorded and uploaded, and then, the participant’s health care professional was contacted by a member of the research team if any medication changes or unscheduled asthma-related visits were needed	Asthma	The changes in ACQ^a^ score from baseline to 3 months for MASTERY and usual care groups were 0.01±0.11 and 0.16± 0.09, respectively. No significant difference in lung function was observed
Homko et al, 2007 [39]	Daily monitoring of blood glucose levels, recording insulin levels and episodes of hypoglycemia, and transmission of the measures to the diabetes health network (with health care providers involved in this network) at least 3 times a week	Diabetes	There was no significant difference between the 2 groups’ blood glucose values and HbA1c levels. Significantly more women in the internet group received insulin therapy (31% vs 4%; *P*<.05). There were no significant differences in pregnancy and neonatal outcomes between the 2 groups
Perez-Ferre et al, 2010 [40]	A telemedicine system for the transmission of capillary glucose data and short text messages with weekly professional feedback	Diabetes	There was no difference in maternal metabolic parameters or in pregnancy outcomes
Homko et al, 2012 [41]	Data transfer from patient to practice and practice to patient to send blood glucose and other health data directly to health care providers to receive information or advice from the health care provider via the internet or phone	Diabetes	There were no significant differences between the 2 groups with regard to maternal blood glucose values or infant birth weight
Carral et al, 2015 [42]	Website which allows remote and bidirectional communication between health care professionals and patients with diabetes, offering the patient the possibility of sending blood glucose values, insulin doses, and other health data that can be evaluated remotely by doctors and nurses in an asynchronous manner	Diabetes	There was no significant difference in HbA1c levels. Significantly less insulin treatment and less health care visits in intervention group were observed
Nicholson et al, 2016 [36]	Web lessons, self-tracking of weight and glucose, automated feedback, and access to a message board for peer support	Diabetes	Average gestational weight gain for all participants was 19.9±13.2 lb. There was no statistically significant difference between baseline and 36 weeks of gestation in HbA1c levels
Stockwell et al, 2014 [43]	In total, 5 weekly text messages regarding influenza vaccination and 2 text message appointment reminders (intervention group); invitation for vaccination through the health care provider (control group)	Vaccination	Women in the intervention group were more likely to receive an influenza vaccination (adjusted odds ratio, AOR 1.3, CI 1.003-1.69)
Jordan et al, 2015 [44]	An encouragement message or an encouragement messages plus the opportunity to schedule a reminder	Vaccination	There was no significant increase of the odds of vaccination at follow-up. Significant increase of continued intent to be vaccinated later in the season (AOR 2.1, 95% CI 1.4-3.1)
Yudin et al, 2017 [45]	In total, 2 messages weekly for 4 consecutive weeks reinforcing that the influenza vaccine is recommended for all pregnant women and safe during pregnancy and breastfeeding vs no messages	Vaccination	There was no significant difference between the intervention and control group

^a^ACQ: Asthma Control Questionnaire.

**Table 4:** Quality scores included studies evaluated on effectiveness in review.

Author (year)	Design	Size	Exposure	Outcome	Adjustment	Total
Carral (2015) [42]	2	2	0	2	0	6
Choi (2016) [20]	2	0	0	2	1	5
Evans (2015) [34]	2	2	0	1	2	7
Fujioka (2012) [32]	1	0	0	2	0	3
Herring (2016) [31]	2	2	0	2	2	8
Homko (2007) [39]	2	1	0	1	0	4
Homko (2012) [41]	2	1	0	1	0	4
Jordan (2015) [44]	2	2	0	1	2	7
Moniz (2015) [33]	1	1	0	1	0	3
Nicholson (2016) [36]	2	0	0	2	0	4
Perez-Ferre (2010) [40]	2	1	0	2	0	5
Pollak (2014) [21]	2	0	0	1	0	3
Soltani (2015) [28]	2	0	0	0	0	2
Stockwell (2014) [43]	2	2	1	2	2	9
van Dijk (2016) [35]	2	2	1	2	0	7
Yudin (2017) [45]	2	2	0	2	0	6

## Discussion

### Purpose of This Review

The purpose of this systematic review was to provide information on the usability, defined as feasibility and acceptability, and effectiveness of both mHealth lifestyle and medical apps related to pregnancy in high-income countries.

### mHealth Lifestyle Apps

The results of this review clearly show the feasibility of most lifestyle apps, according to the criteria defined for this review. After activation, there is an adequate short- and long-term use as well as intention to use these apps [17-25]. This is in line with the perceived suitability of the apps that are often judged as good, easy, and simple to use. Moreover, the lifestyle apps with a target on improvement of health behavior, less gestational weight gain, and smoking cessation showed positive results on effectiveness. However, due to small sample size, significances could often not be demonstrated [21-23,27,28,31-35]. These results are in line with the systematic review by Badawy et al, which evaluated texting and apps for preventive behavior in adolescents [46]. They concluded that most studies reported positive on feasibility with high acceptability and satisfaction. This review included studies focusing on clinic attendance, contraceptive use, oral health, physical activity and weight management, sun protection, human papillomavirus vaccination, smoking cessation, and sexual health.

We observed high dropout rates among users of several apps [17,18,20]. This is in line with qualitative research by Dennison et al who found that participants lacked commitment using any particular app and seemed likely to engage in only transient, casual use [47]. These findings could be of concern for apps that aim to support long-term lifestyle interventions. For pregnancy apps, this is not necessarily a barrier as the use of these apps is narrowed by a limited time frame.

We were surprised to find only 1 study involving also male partners in the intervention of adopting healthy nutrition and lifestyle [35]. This study clearly showed that women whose partners also participated showed the strongest positive change of these behaviors, which was significantly associated with a higher chance of achieving a pregnancy (adjusted hazard ratio 0.75, 95% CI 0.61-0.91).

### mHealth Medical Apps

In contrast to the mHealth lifestyle apps, the feasibility and acceptability of the medical apps was only reported in 2 studies concerning diabetes management and judged as good [36,37]. The evaluation of 5 studies on effectiveness of diabetes treatment during pregnancy could be judged properly because of objective outcome measures.

The effectiveness of mHealth medical apps in improving asthma management [38] and vaccination rates [43-45] is promising. However, not all studies showed significant outcomes, due to small sample sizes.

The study by Nes et al found that their mobile app for self-management of type 2 diabetes is feasible because of a high response rate. The intervention was evaluated as supportive and meaningful [48]. Hayashi et al tested the feasibility and usability of a self-management support system for dialysis patients and concluded that the completion rate was good, and most patients appreciated the system and intended to continue using the system [49].

Ming et al [50] evaluated 7 randomized controlled trials on telemedicine in gestational diabetes in a meta-analysis. A modest but statistically significant improvement in HbA1c associated with the use of a telemedicine technology was demonstrated; however, there was insufficient evidence that other clinical endpoints were affected. In agreement with our results and due to lack of trials with large sample size and the variations of technologies used, it is not possible to draw a strong conclusion on the genuine benefits of the apps.

### Combination of mHealth Lifestyle and Medical Apps

Rehman et al reviewed the literature on the combination of mHealth apps involving smoking cessation and general diabetes management [51]. The authors reported low absolute smoking cessation rates, even though in some studies, intervention groups performed better than controls. They showed that mHealth could play a potential role in diabetes management; for example, text messages showed mixed results on HbA1c levels, which is in line with our results [36,39-41].

### Strength and Limitations

The main strength of this systematic review is that we evaluated both the feasibility and acceptability as well as the effectiveness of mHealth apps with a focus on lifestyle and medical domains. We limited our systematic review to apps for high-income countries and have made this choice because the needs and populations are very different between high- and low-middle-income countries. Hence, a broad view is given of existing evidence on factors influencing the implementation of new mHealth apps. The field of mHealth is fast growing with increasing evidence to support benefits for patients improving health outcomes as well as quality of health care. Furthermore, we used a systematic search method assisted by a clinical librarian.

Our study has also some limitations. The qualitative studies fail quality assessment using the predefined quality assessment (COREQ) [16]. With regard to the interpretation and validity of the results, we encountered a poor quality of most studies due to small sample sizes, high dropout rates after randomization for unknown reasons (cave selection bias), and the use of subjective outcome measures [23,40].

Therefore, an overestimation of the outcomes is very likely because it is known that, in particular, motivated women most often apply and continue the use of the intervention. Another issue of concern is the lack of objectivity of the data in most studies, because of the self-reporting of questionnaire data.

We did not include “adherence to medication” and “security” in our search. However, it is very worthwhile to address the systematic review by Badawy et al, showing the feasibility, acceptability, and efficacy of mHealth apps to improve adherence to medication use in adolescents with chronic health conditions [52]. The barriers of security and privacy issues of mHealth technology are not often addressed. Kotz et al [53] is warning about the fact that many health care organizations lack the technology and expertise to secure patient data for cyberattacks in medical devices. Only the study by Hirst et al reported on a secured website for communication and transmission of confidential data [37]. It is possible that security issues influence the feasibility and acceptability as well as the effectiveness of mHealth apps. This raises the discussion whether the quality of these mHealth apps developed for health care have to be certified, such as a Conformité Européenne (CE, meaning European Conformity) certification. The advantage will be that the quality of all apps will be controlled and improved and the implementation of poor-quality apps will be limited.

### Conclusion and Practical Implications

This review outlines that most mHealth lifestyle and medical apps for pregnant women seem to be feasible and acceptable. mHealth crosses the boundaries of many related health fields, such as pediatrics, internal medicine, and social medicine. Therefore, future research should also focus on the impact of mHealth on related health conditions, clinical practice, and cost-effectiveness. This is supported by Badawy et al, showing that there is plenty room for further research in particular with regard to cost savings of mHealth by improving, eg, adherence to medication use [54].

We found modest evidence on effectiveness because most intervention studies evaluated small study groups, resulting in only a tendency toward positive results in the intervention groups and rarely significant improvements. We recommend that the development as well as the examination of feasibility and acceptability of new mHealth apps for (maternal) health care and lifestyle support should be done together with the target group. A clear definition of feasibility and acceptability within the focus of the app must be maintained as, for example, maternity care asks other definitions as antenal care.

Finally, we and others are convinced that it is necessary to thoroughly guarantee security and privacy of the mHealth apps used in health care and beyond. Therefore, we strongly plea for the development of formal guidelines for quality certification of the apps before introduction.

## Appendix

Multimedia Appendix 1 Search strategy.

Multimedia Appendix 2 Quality score for systematic reviews.

Multimedia Appendix 3 Study characteristics of mHealth lifestyle apps.

Multimedia Appendix 4 Study characteristics of mHealth medical apps.

Multimedia Appendix 5 Complete overview of results on mHealth lifestyle apps.

Multimedia Appendix 6 Overview of studies reporting on lifestyle mHealth apps: feasibility.

Multimedia Appendix 7 Overview of studies reporting on lifestyle mHealth apps: acceptability.

Multimedia Appendix 8 Overview of studies reporting on lifestyle mHealth apps: effectiveness.

Multimedia Appendix 9 Complete overview of results on mHealth medical apps.

## Abbreviations

COREQ: consolidated criteria for reporting qualitative research
GDM: gestational diabetes mellitus
mHealth: mobile health
SMS: short message service
