# Supplementary material for: The Usability and Effectiveness of Mobile Health Technology–Based Lifestyle and Medical Intervention Apps Supporting Health Care During Pregnancy: Systematic Review
Source: JMIR Mhealth Uhealth. 2018 Apr 24;6(4):e109. doi: 10.2196/mhealth.8834 (PMC5941088; doi:10.2196/mhealth.8834)
Supplement: Multimedia Appendix 1 [file mhealth_v6i4e109_app1.pdf]

Appendix 1. Search strategy.

Date of search: February 6, 2017

Sources: Embase.com, Medline (Ovid), Cochrane Central, Web of Science, PubMed recent, Google scholar.

Database	*Number of refs*	*Refs after deduplication*
Embase.com	1423	1380
Medline Epub (Ovid)	1270	527
Cochrane Central	186	8
Web of Science	1125	455
Google Scholar	200	117
Total	***4204***	***2487***

Results

Removed duplicates: 1717

Update Feb 15th 2016 – Feb 6th 2017: 583

Embase.com (Embase incl. Medline): 1423

('mobile application'/de OR 'telehealth'/de OR 'mobile phone'/de OR 'text messaging'/de OR ('e-health' OR ehealth OR mhealth OR 'm-health' OR telehealth OR ((tele OR mobile) NEAR/3 health) OR smartphone* OR ((mobile OR smart OR Android OR iOS) NEAR/3 (phone* OR monitor* OR device*)) OR (mobile NEXT/1 based) OR 'm-phone' OR mphone* OR 'cell phone' OR iphone OR ipad OR ipod OR ((app OR apps OR application*) NEAR/3 (mobile OR portable OR tablet OR Android OR iOS OR web OR online OR communication OR customized)) OR (text NEAR/3 messag*) OR SMS):ab,ti**) AND (**'pregnancy'/exp OR 'pregnant woman'/exp OR 'pregnancy outcome'/exp OR 'pregnancy complication'/exp OR 'morning sickness'/de OR 'perinatal care'/exp OR 'obstetrics'/exp OR 'prepregnancy care'/de OR 'fetus'/exp OR 'fetus outcome'/de OR 'fetus death'/de OR 'fetus mortality'/de OR 'newborn death'/de OR 'maternal mortality'/de OR 'maternal disease'/exp OR (obstetric* OR pregnan* OR gestation* OR childbearing* OR (child NEAR/3 bearing) OR gravid* OR maternal* OR maternity OR mother* OR fetal* OR foetal OR fetus OR foetus OR prepregnan* OR preconception* OR antenatal* OR prenatal* OR perinatal* OR (morning NEXT/1 sickness) OR eclampsia* OR stillbirth*):ab,ti**) NOT (**[Animals]/lim NOT Humans]/lim**)**

Medline (Ovid): 1270

("Mobile Applications"/ OR "TeleMedicine"/ OR exp "Cell Phones"/ OR "Text Messaging"/ OR ("e-health" OR ehealth OR mhealth OR "m-health" OR telehealth OR ((tele OR mobile) ADJ3 health) OR smartphone* OR ((mobile OR smart OR Android OR iOS) ADJ3 (phone* OR monitor* OR device*))

OR (mobile ADJ1 based) OR "m-phone" OR mphone* OR 'cell phone' OR iphone OR ipad OR ipod OR ((app OR apps OR application*) ADJ3 (mobile OR portable OR tablet OR Android OR iOS OR web OR online OR communication OR customized)) OR (text ADJ3 messag*) OR SMS).ab,ti.**) AND (**exp "Pregnancy"/ OR "Pregnant Women"/ OR exp "Pregnancy Complications"/ OR "Prenatal Care"/ OR "Perinatal Care"/ OR "Obstetrics"/ OR "Preconception Care"/ OR exp "Fetus"/ OR "Maternal Health"/ OR "Maternal Mortality"/ OR (obstetric* OR pregnan* OR gestation* OR childbearing* OR (child ADJ3 bearing) OR gravid* OR maternal* OR maternity OR mother* OR fetal* OR foetal OR fetus OR foetus OR prepregnan* OR preconception* OR antenatal* OR prenatal* OR perinatal* OR (morning ADJ1 sickness) OR eclampsia* OR stillbirth*).ab,ti.**) NOT (**animals NOT humans**)**.sh.

Cochrane Central (trials): 186

(('e-health' OR ehealth OR mhealth OR 'm-health' OR telehealth OR ((tele OR mobile) NEAR/3 health) OR smartphone* OR ((mobile OR smart OR Android OR iOS) NEAR/3 (phone* OR monitor* OR device*)) OR (mobile NEXT/1 based) OR 'm-phone' OR mphone* OR 'cell phone' OR iphone OR ipad OR ipod OR ((app OR apps OR application*) NEAR/3 (mobile OR portable OR tablet OR Android OR iOS OR web OR online OR communication OR customized)) OR (text NEAR/3 messag*) OR SMS):ab,ti**) AND (**(obstetric* OR pregnan* OR gestation* OR childbearing* OR (child NEAR/3 bearing) OR gravid* OR maternal* OR maternity OR mother* OR fetal* OR foetal OR fetus OR foetus OR prepregnan* OR preconception* OR antenatal* OR prenatal* OR perinatal* OR (morning NEXT/1 sickness) OR eclampsia* OR stillbirth*):ab,ti**)**

Web of Science: 1125

TS=(("e-health" OR ehealth OR mhealth OR "m-health" OR telehealth OR ((tele OR mobile) NEAR/2 health) OR smartphone* OR ((mobile OR smart OR Android OR iOS) NEAR/2 (phone* OR monitor* OR device*)) OR (mobile NEAR/1 based) OR "m-phone" OR mphone* OR "cell phone" OR iphone OR ipad OR ipod OR ((app OR apps OR application*) NEAR/2 (mobile OR portable OR tablet OR Android OR iOS OR web OR online OR communication OR customized)) OR (text NEAR/2 messag*) OR SMS**) AND (**obstetric* OR pregnan* OR gestation* OR childbearing* OR (child NEAR/2 bearing) OR gravid* OR maternal* OR maternity OR mother* OR fetal* OR foetal OR fetus OR foetus OR prepregnan* OR preconception* OR antenatal* OR prenatal* OR perinatal* OR (morning NEAR/1 sickness) OR eclampsia* OR stillbirth***) NOT ((**animal* OR rat OR rats OR mice OR mouse OR rabbit* OR plant) NOT (human*)**))**

Google Scholar: 200 *(top relevance)*

"e health"|ehealth|telehealth|"tele health"|"mobile|portable|tablet app|application|apps|device|phone|device"|"text messaging" pregnancy|pregnant|gestation|childbearing|maternal|mother|maternity|preconception
