# Supplementary material for: The Usability and Effectiveness of Mobile Health Technology–Based Lifestyle and Medical Intervention Apps Supporting Health Care During Pregnancy: Systematic Review
Source: JMIR Mhealth Uhealth. 2018 Apr 24;6(4):e109. doi: 10.2196/mhealth.8834 (PMC5941088; doi:10.2196/mhealth.8834)
Supplement: Multimedia Appendix 2 [file mhealth_v6i4e109_app2.pdf]

Appendix 2. Quality score for systematic reviews

ErasmusAGE, 24 June 2013

This quality score can be used to assess the quality of studies included in systematic reviews and meta-analyses and is applicable to both interventional and observational studies. The score was designed based on previously published scoring systems (Carter et al., 2010 and the Quality Assessment Tool for Quantitative Studies). The quality score is composed of 5 items, and each item is allocated 0, 1 or 2 points. This allows a total score between 0 and 10 points, 10 representing the highest quality.
The version presented below is a general version and needs to be adapted for each review separately , e.g. concerning what study size is large or small within the study field, what exposure and outcome measurement methods are adequate, and what the key confounders are. Decisions on these detailed criteria should be based on literature, guidelines and/or discussions with experts. The criteria should be defined before the review process.

Study design

0 for studies with cross-sectional data collection

1 for studies with longitudinal data collection (both retrospective and prospective)

2 for intervention studies

Study size (predefined) *

0 small population for analysis

1 intermediate population for analysis

2 large population for analysis

Exposure

Observational studies

0 if the study used no appropriate exposure measurement method or if not reported

1 if the study used moderate quality exposure measurement methods

2 if the study used adequate exposure measurement methods*

Intervention studies

0 if the intervention was not described or not blinded

1 if the intervention was adequately single blinded.

2 if the intervention was adequately double-blinded.

Outcome

0 if the study used no appropriate outcome measurement method or if not reported

1 if the study used moderate quality outcome measurement methods

2 if the study used adequate outcome measurement methods*

Adjustments

0 if findings are not controlled for at least key confounders† *

1 if findings are controlled for key confounders

2 if findings are additionally controlled for additional covariates or when an intervention is adequately randomized

* Needs to be specified for each review, based on literature, guidelines and/or expert opinions in the field

† Either adjusted for in the statistical analyses; stratified for in the analyses; or not applicable (e.g. a study in women only does not require controlling for sex)
