# Supplementary material for: The Usability and Effectiveness of Mobile Health Technology–Based Lifestyle and Medical Intervention Apps Supporting Health Care During Pregnancy: Systematic Review
Source: JMIR Mhealth Uhealth. 2018 Apr 24;6(4):e109. doi: 10.2196/mhealth.8834 (PMC5941088; doi:10.2196/mhealth.8834)
Supplement: Multimedia Appendix 3 [file mhealth_v6i4e109_app3.pdf]

Appendix 3. Study characteristics of mHealth lifestyle applications

**Author (year) location**	**Subject**	**Study type**	**N (intervention, control)**	**Intervention (name of intervention)**	**Gestational age (GA) at start intervention and mean GA at enrollment**
van Zutphen (2008) The Netherlands	Fruit, vegetables, smoking, folic acid, alcohol	Survey	408*	eHealth program	All gestational ages
Bot (2009) The Netherlands	Fruit, vegetables, smoking, folic acid, alcohol	Prospective cohort	13946*	Email-based health promotion program.	All gestational ages
Dalrymple (2013) USA	Fruit, vegetables, smoking, folic acid, alcohol	Survey	31	MHealth program for behavior change - SMS (Text4baby)	NA (~)
Song (2013) USA	Vitamins, morning sickness, foods, abdominal pains	Pilot	23	Two-way text messaging system (automatic response system)	Mean GA 22.5 weeks ± 5.08
Gazmararian (2014) USA	Fruit, vegetables, physical activity	Prospective cohort	83*	MHealth program for behavior change - SMS (Text4baby)	NA (~)
Evans (2015) USA	Fruit, vegetables, prenatal vitamins, smoking, alcohol	RCT^	943 (473^,470)	MHealth program for behavior change - SMS (Text4baby)	Before 14 weeks of gestation. Mean GA 8.1 weeks ± 1.9

van Dijk (2016) The Netherlands	Fruit, vegetables, smoking, folic acid, alcohol	Survey	1878	Web-based Smarter Pregnancy Platform	Couples contemplating pregnancy or pregnant couples
Pollak (2014) USA	Weight control	RCT (pilot) and feasibility study	35 (23,12)*	SMS intervention (PregChat) vs generic texting intervention (Text4Baby)	Between 12-21 weeks of gestation, mean GA at baseline 16 weeks
Knight-Agarwall (2015) Australia	Weight control	Mixed method: Survey, focus group and interview	10	An app with gestational weight gain calculator, general dietary information and motivation (Eating4Two)	Before 30 weeks of gestation, range between 13-30 weeks
Soltani (2015) UK	Weight control	Intervention and feasibility study	29 (14,15)	Text message based intervention program (incl. 4 visits en food and activity daybook (MOMTech)	Before 14 weeks of gestation
Herring (2016) USA	Weight control	RCT	66 (33,33)	Technology-based behavioral weight control intervention - SMS, Facebook, telephonic counseling	Before 20 weeks of gestation. Mean GA 13.4 weeks ± 4.1 (usual care), 11.5 weeks ± 2.9 (intervention)

Choi (2015) USA	Physical activity	RCT	30 (15,15)	Mobile phone application as addition to Fitbit ultra	Between 10 and 20 weeks of gestation
Fujioka (2012) Japan	Smoking cessation	Prospective cohort	66	Mobile phone e-learning smoking cessation support program	Over 20 weeks of gestation
Naughton (2012) UK	Smoking cessation	RCT and feasibility study	207 (102,105)	Automated text messaging based smoking cessation intervention (MiQuit)	Before 21 weeks of gestation. Mean GA 12.7 ± 3.3 (intervention), 12.8 ± 3.2 (controls)
Naughton (2013) UK	Smoking cessation	Semi-structured interview and focus group	13*	Automated text messaging based smoking cessation intervention (MiQuit)	NA (~)
Pollak (2013) USA	Smoking cessation	RCT and feasibility study	31 (15,16)	SMS support vs SMS support and Scheduled Gradual Reduction (SGR)	Between 10-30 weeks gestation
Davis (2014) USA	Smoking cessation, mental health	Survey<br>Pilot	68<br>5	E-health educational intervention on health behaviors	NA (~)
Abroms (2015) USA	Smoking cessation	Survey	20	Proven smoking-cessation text	Before 30 weeks of gestation

				message based program (Quit4baby)	
Moniz (2015) USA	Smoking cessation, nutrition	Prospective cohort	171	Text message based intervention	Before 28 weeks of gestation. Mean GA 15.7 ±6.2 SD

*analysis of the pregnant women in this cohort
(~) NA= not available
