# Supplementary material for: The Usability and Effectiveness of Mobile Health Technology–Based Lifestyle and Medical Intervention Apps Supporting Health Care During Pregnancy: Systematic Review
Source: JMIR Mhealth Uhealth. 2018 Apr 24;6(4):e109. doi: 10.2196/mhealth.8834 (PMC5941088; doi:10.2196/mhealth.8834)
Supplement: Multimedia Appendix 4 [file mhealth_v6i4e109_app4.pdf]

Appendix 4. Study characteristics of mHealth medical applications

**Author (year) location**	**Subject**	**Study type**	**N (intervention, control)**	**Intervention (name of intervention)**	**Gestational age (GA) at start intervention and mean GA at enrollment if applicable.**
Zairina (2015) Australia	Asthma	RCT	72 (36,36)	Management of Asthma with Supportive Telehealth of Respiratory function in pregnancy (MASTERY) vs usual care	Before 20 weeks of gestation. Mean GA 17.7 weeks ± 3.1
Carral (2015) Spain	Diabetes	CT(^) (self-selected)	104 (40,64)	Web-based telemedicine monitoring system (DiabeTIC)	Before 30 weeks of gestation. Mean GA 21.1 weeks ± 9.6
Homko (2012) USA	Diabetes	RCT	80 (40,40)	Web-based nurse-coordinated communicatio n system	Before 33 weeks of gestation. Mean GA 28.4 weeks ± 3.4 (controls), 28.5 ±4.2 (telemedicine)
Homko (2007) USA	Diabetes	RCT	63 (34,29)	Telemedicine system	Before 33 weeks of gestation. Mean GA 27.7 weeks ± 3.8 (controls), 27.5 ±4.2

					(telemedicine)
Hirst (2015) UK	Diabetes	Survey	52	Interactive blood glucose management system	Before 34 weeks of gestation
Nicholson (2016) USA	Diabetes	Single-arm pretest-posttest	23	Web-based pregnancy and postpartum lifestyle intervention (GooDMomS)	Mean GA at baseline 28.9 ± 3.4 weeks
Perez-Ferre (2010) Spain	Diabetes	RCT	100 (50,50)	Telemedicine system with a glucometer, application and SMS	Before 28 weeks of gestation
Stockwell (2014) USA	Influenza vaccination	RCT	1187 (593,594)	Automated text message based intervention of influenza vaccine reminders	All gestational ages
Jordan (2015) USA	Influenza vaccination	RCT*^	Total: 16830 Planners: 5024(2468,2556) Non-planners 5292(2666,2626)	Four interventions: Planners: 1= an encouragement message 2= an encouragement message plus the opportunity to schedule a reminder.	All gestational ages

				Non-planners: 3= receive general education 4= education tailored to their reason for non-vaccination.	
Yudin (2017) Canada	Influenza vaccination	RCT	317 (153,164)	Text message based intervention (twice weekly for four weeks)	All gestational ages. Mean GA 22 weeks

*Analysis of the pregnant women in this cohort
^More than one intervention
~Several types of diabetes combined
(*)Salivary validated seven consecutive days of non-smoking
(^)CT= Controlled trial
(~)NA= not available
