# Supplementary material for: The Usability and Effectiveness of Mobile Health Technology–Based Lifestyle and Medical Intervention Apps Supporting Health Care During Pregnancy: Systematic Review
Source: JMIR Mhealth Uhealth. 2018 Apr 24;6(4):e109. doi: 10.2196/mhealth.8834 (PMC5941088; doi:10.2196/mhealth.8834)
Supplement: Multimedia Appendix 5 [file mhealth_v6i4e109_app5.pdf]

*Appendix 5. Complete overview of results on mHealth lifestyle applications*

| Author year | Focus | Usability | | | | Acceptability | | | Effectiveness |
|---|---|---|---|---|---|---|---|---|---|
| | | **Actual use** | **Interest** | **Appropriateness** | **Ability** | **User satisfaction** | **Appreciation** | **Recommendation** | |
| van Zutphen 2008 | Fruit, vegetables, smoking, folic acid, alcohol | 59% of sent quiz emails were opened and of those 85% of the questions were accessed. 37% of the practical tips 12% of links to related websites were used. | | | 96% of respondents (n=163) evaluated the program as easy to understand | | 81% of respondents (n=163) evaluated the program as reliable. | | |
| Bot 2009 | Fruit, vegetables, smoking, folic acid, alcohol | 16% of participants opened all quiz emails received. 18% of participants never opened a quiz email. 27% of participants ceased participation. | | | | | 78% of the respondents (n=3763) showed a high or intermediate appreciation. | | |
| Dalrymple 2013 | Fruit, vegetables, smoking, folic acid, alcohol | | Many participants expressed an interest in continuing to seek health information on the internet. | | | All respondents found the program was "very" or "somewhat" helpful (n=7). | All respondents found the program was "very" or "somewhat" interesting (n=7). | | |
| Song 2013 | Vitamins, morning sickness, | | | 65% agreed/strongly agreed that the | 80% agreed or strongly agreed that it was easy to | | | | |

| | | | | | | | | | |
|---|---|---|---|---|---|---|---|---|---|
| | foods, abdominal pains | | | information was easy to understand. | text their questions. 65% agreed or strongly agreed using the text message service made finding answers quick and easy. | | | | |
| Gazmararian 2014 | Fruit, vegetables, physical activity | 95 % of the respondents (n=50) regularly read all messages throughout the study. | 88 % of the respondents (n=50) planned to continue being enrolled. | | 96% of the respondents (n=50) reported receiving messages without interruption. | | | | |
| van Dijk 2016 | Fruit, vegetables, smoking, folic acid, alcohol | | | | | | | | Effectiveness on all patients: Vegetable intake: improvement of 20.9% (6 weeks) and increase up to 26.3% (6 months) - Fruit intake improvement by 36.1 and 38.4% (6 weeks and 6 months), - Inadequate folic acid use: decrease of 53.6% and 56.3%, - Tobacco and alcohol use were reduced by 23.8% and 27.0% at 6 weeks and 6 months. |
| Evans 2015 | Fruit, vegetables, prenatal vitamins, smoking, alcohol | | | | | | | | No significant difference in health behavior between intervention and controls. Significant lower self-reported alcohol consumption in high dosage intervention group. |
| Pollak 2014 | Weight control | 86% of the respondents in the PregCHAT text arm reported reading their texts and responded to their texts. | | | | Women in the PregCHAT text arm rated the program as helpful (64% rated 5 or higher on a 7-point Likert item) | | 80 % of the women in the PregCHAT text arm would recommend the program to a friend. | There was a non-significant difference in mean gestational weight gain 6 pounds less for women who completed the intervention, between the PregCHAT arm and the Txt4Baby arm. No differential treatment effect was found in self-reported physical activity or the nutrition score. |

| | | | | | | | | | | |
|---|---|---|---|---|---|---|---|---|---|---|
| Knight-Agarwall 2015 | Weight control | | Most women expressed desire to continue using the application. | | Women found the application generally simple to use. | 70% found the weight gain graph helpful. 80% found the nutrition advise helpful or very helpful. | Participants enjoyed using the application. | | | |
| Soltani 2015 | Weight control | | Some participants suggested continuing the intervention postnatally | | | | Participants liked the holistic nature of the intervention. | | Mean GWG; 6.65 in intervention group vs 9.74 in controls. (NB: No statistical analysis were performed due to small sample size) | |
| Herring 2016 | Weight control | | | | | 96% reported that the skills they learned in the program were extremely helpful (at least an 8 on a 10-point scale); 96% found the text messages extremely useful. 82% found the coach calls extremely useful. 87% reported the program was extremely successful in changing eating habits. | | | Weight gain was significantly reduced in the intervention group compared to usual care (37% vs. 66%, P 0.033). No significant differences in neonatal or obstetric outcomes were found. | |
| Choi 2015 | Physical activity | 78% response rate of daily messages at two weeks participation | Response rate dropped to 24% at ten weeks of participation. | | | | | | Intervention participants tend to increase in daily steps compared to control participants but not significantly different. | |

| | | | | | | | | | |
|---|---|---|---|---|---|---|---|---|---|
| Fujioka 2012 | Smoking cessation | | | | | | | | The rate of non-smoking was 71.1% of participants who completed the study (n=48). Carbon monoxide exhalation levels significantly decreased (from 6.43 +/- 4.5 ppm at the beginning to 0.7 +/- 1.0 ppm in 1 month, to 0.29 +/- 1.08 in 3 months (P < 0.001). |
| Naughton 2012 | Smoking cessation | Of the MiQuit participants, the response rates to assessment text messages sent: 3 week smoking text message: 64% of the intervention arm, at 7 weeks: 51%. | 9% of the MiQuit participants discontinued the texts. | 24% of the MiQuit participants thought the texts were annoying. 26% felt they had received too many messages. | | Between 81% and 96% of the MiQuit participants rated the messages as helpful to some degree. | | | At 3-month follow-up, there was no statistically significant difference between intervention and controls. (NB: the trial was not powered to detect a group difference on smoking outcomes) |
| Naughton 2013 | Smoking cessation | | Participants felt they would read all the texts. | Participants did not feel the approach of the intervention was inappropriate. | Participants think the tool is highly convenient. | | | | |
| Pollak 2013 | Smoking cessation | 86% of women in both groups reported reading “all or most” of the support messages. Women in the SGR arm respond to 68% of alert texts within 60 min. | | | | Women found the program helpful (mean score of 6 out of 7 in a 7-point scale). | Women found the program to be acceptable (mean score of 6 out of 7 in a 7-point scale) | Most women would recommend the program to a friend, with 78% in both arms reporting 7 in a 7-point scale. | The intervention group tends to a lower prevalence of smoking: 7-day point prevalence of 13.4% versus 7.5% in favour of the SGR arm. (NB: given the small sample size, the purpose of the arms is simply to monitor for unexpected, gross differences between arms) |
| Davis 2014 | Smoking cessation, mental health | All participants read all text messages once or twice. (n=5) | 66% agreed and strongly agreed they were interested in the service. | | The technology was user friendly. (n=5) | All participants found the text messages useful. (n=5) | | | |

| | | | | | | | | | |
|---|---|---|---|---|---|---|---|---|---|
| Abroms 2015 | Smoking cessation | All respondents have read all the text messages (n=16). | | Received number of text messages was "just right" (n=14) or "too few" (n=2). | | All respondents (n=16): "agreed" and "completely agreed" that the program was helpful in quitting. | Most participants liked the program overall. | All respondents (n=16) would recommend the program to a friend. | |
| Moniz 2015 | Smoking cessation, nutrition | | | | | | | | Improvements in self-reported behaviour between baseline and follow-up: decreased tobacco use in 41% of the smokers (n=44). More consistent condom use in 7% of the sexually active participants (n=83). 32% more prenatal vitamin intake. 32% more frequent seatbelt use. 41% more frequent healthy food intake. |
