# Supplementary material for: The Usability and Effectiveness of Mobile Health Technology–Based Lifestyle and Medical Intervention Apps Supporting Health Care During Pregnancy: Systematic Review
Source: JMIR Mhealth Uhealth. 2018 Apr 24;6(4):e109. doi: 10.2196/mhealth.8834 (PMC5941088; doi:10.2196/mhealth.8834)
Supplement: Multimedia Appendix 6 [file mhealth_v6i4e109_app6.pdf]

Multimedia Appendix 6. Overview of studies reporting on lifestyle mHealth apps: feasibility.

**Author year**	**Technique**	**Focus**	**Feasibility**			
			**Actual use**	**Interest**	**Suitability**	**Ability**
van Zutphen et al 2008 [17]	Email containing a quiz with a maximum of six questions, tailored to the stage of pregnancy on health related topics (nutrition, exercise, lifestyle, smoking, safety)	Fruit, vegetables, smoking, folic acid, alcohol	59% of sent quiz emails were opened and of those 85% of the questions were accessed. 37% of the practical tips 12% of links to related websites were used.			96% of respondents (n=163) evaluated the program as easy to understand
Bot et al 2009 [18]	Emails containing quizzes on the topics: nutrition, smoking, physical activity, safety, lifestyle or care and pregnancy every 4 weeks	Fruit, vegetables, smoking, folic acid, alcohol	16% of participants opened all quiz emails received. 18% of participants never opened a quiz email. 27% of participants ceased participation.			
Dalrymple et al 2013 [19]	Text messaging three times a week until a baby is 1 year of age; based on topics discussed in prenatal classes	Fruit, vegetables, smoking, folic acid, alcohol		Many participants expressed an interest in continuing to seek health information on the internet		

Song et al<br><br>2013 [20]	Use of TuTalk, an existing system for two-way, text based dialog for question and answer	Vitamins morning sickness foods abdominal pains			65% agreed or strongly agreed that the informati on was easy to understan d	80% agreed or strongly agreed that it was easy to text their questions . 65% agreed or strongly agreed using the text message service made finding answers quick and easy.
Gazmara rian et al<br><br>2014 [21]	Three weekly messages on nutrition and physical activity targeted to the weeks along in pregnancy or the age (in weeks) of their child.	Fruit, vegetables , physical activity	95% of the respondents (n=50) regularly read all messages throughout the study.	88% of the respondent s (n=50) planned to continue being enrolled.		96% of the responde nts (n=50) reported receiving messages without interrupti on
Pollak et al<br><br>2014 [23]	SMS- texting intervention (PregCHAT) vs a generic texting intervention TexT4baby):PregC HAT = Personalized feedback based on women's intake of sweetened beverages, fruits and vegetables, fast food, daily	Weight control	86% of the respondents in the PregCHAT text arm reported reading their texts and responded to their texts.			

	steps taken and weight<br>TexT4baby = general information to improve health					
Knight-Agarwall et al<br>2015 [24]	Eating4Two app which provided a graphing function to record the weight through the pregnancy with real-time feedback on weight gain and general information on antenatal nutrition	Weight control		Most women expressed desire to continue using the application		Women found the application generally simple to us
Soltani et al<br>2015 [25]	MOMTech: Text messages twice a day including self-monitoring weight management and feedback on setting goals	Weight control		Some participants suggested continuing the intervention postnatally.		
Choi et al<br>2015 [22]	SMS-texting intervention; daily messages and a mobile phone activity diary with automated feedback and self-monitoring systems	Physical activity	78% response rate of daily messages at two weeks participation.	Response rate dropped to 24% at ten weeks of participation		
Naughton et al 2012 [26]	Tailored self-help leaflet followed by an 11-week program of tailored text messages or (controls) a nontailored self-help leaflet	Smoking cessation	Of the MiQuit participants, the response rates to assessment text messages sent: 3 week smoking text message: 64%	Nine percent of the MiQuit participants discontinued the texts.	24% of the MiQuit participants thought the texts were annoying	

			of the intervention arm, at 7 weeks: 51%.		. 26% felt they had received too many messages .	
Naughton et al 2013 [27]	Tailored self-help leaflet followed by an 11-week program of tailored text messages or (controls) a nontailored self-help leaflet	Smoking cessation		Participants felt they would read all the texts.	Participants did not feel the approach of the intervention was inappropriate.	Participants think the tool is highly convenient.
Pollak et al 2013 [28]	SMS-delivered support messages or support messages plus a scheduled gradual reduction (SGR)	Smoking cessation	86% of women in both groups reported reading "all or most" of the support messages. Women in the SGR arm responded to 68% of alerts texts within 60 min.			
Davis et al 2014 [29]	Daily text messages and three 20-min voice-over-Powerpoint weekly	Smoking cessation, mental Health	All participants read all text messages once or twice (n=5).	66% agreed and strongly agreed they were interested in the service.		The technology was user friendly (n=5).
Abroms et al 2015 [30]	Either receiving sms-text messaging for the TexT4baby program to	Smoking cessation	All respondents have read all the text messages		Received number of text messages was "just	

	improve health or receiving sms-text messaging for the TexT4baby program as well as Quit4Baby program which aimed to improve self-efficacy for smoking cessation		(n=16).		right" (n=14) or "too few" (n=2).	

References

17. van Zutphen M, Milder IE, Bemelmans WJ. Usage of an online healthy lifestyle program by pregnant women attending midwifery practices in Amsterdam. Prev Med. 2008 Jun;46(6):552-7. PMID: 18302971. doi: S0091-7435(08)00006-6.[pii] 10.1016/j.ypmed.2008.01.003.

18. Bot M, Milder IE, Bemelmans WJ. Nationwide implementation of Hello World: a Dutch email-based health promotion program for pregnant women. J Med Internet Res. 2009 Jul 30;11(3):e24. PMID: 19674957. doi: v11i3e24.[pii] 10.2196/jmir.1183.

19. Dalrymple PW, Rogers M, Zach L, Turner K, Green M. Collaborating to develop and test an enhanced text messaging system to encourage health information seeking. J Med Libr Assoc. 2013 Jul;101(3):224-7. PMID: 23930095. doi: 10.3163/1536-5050.101.3.014 JMLA-D-12-00077.[pii]

20. Song H, May A, Vaidhyanathan V, Cramer EM, Owais RW, McRoy S. A two-way text-messaging system answering health questions for low-income pregnant women. Patient Educ Couns. 2013 Aug;92(2):182-7. PMID: 23711635. doi: S0738-3991(13)00174-2.[pii] 10.1016/j.pec.2013.04.016.

21. Gazmararian JA, Elon L, Yang B, Graham M, Parker R. Text4baby program: an opportunity to reach underserved pregnant and postpartum women? Matern Child Health J. 2014 Jan;18(1):223-32. PMID: 23494485. doi: 10.1007/s10995-013-1258-1.

22. Choi J, Lee JH, Vittinghoff E, Fukuoka Y. mHealth Physical Activity Intervention: A Randomized Pilot Study in Physically Inactive Pregnant Women. Matern Child Health J. 2016 May;20(5):1091-101. PMID: 26649879. doi: 10.1007/s10995-015-1895-7 10.1007/s10995-015-1895-7.[pii]

23. Pollak KI, Alexander SC, Bennett G, Lyna P, Coffman CJ, Bilheimer A, et al. Weight-related SMS texts promoting appropriate pregnancy weight gain: a pilot study. Patient Educ Couns. 2014 Nov;97(2):256-60. PMID: 25153313. doi: S0738-3991(14)00304-8.[pii] 10.1016/j.pec.2014.07.030.

24. Knight-Agarwal C, Davis DL, Williams L, Davey R, Cox R, Clarke A. Development and Pilot Testing of the Eating4two Mobile Phone App to Monitor Gestational Weight Gain. JMIR Mhealth Uhealth. 2015 Jun 05;3(2):e44. PMID: 26048313. doi: v3i2e44.[pii] 10.2196/mhealth.4071.

25. Soltani H, Duxbury AM, Arden MA, Dearden A, Furness PJ, Garland C. Maternal Obesity Management Using Mobile Technology: A Feasibility Study to Evaluate a Text Messaging Based

Complex Intervention during Pregnancy. J Obes. 2015;2015:814830. PMID: 25960889. doi: 10.1155/2015/814830.

26. Naughton F, Prevost AT, Gilbert H, Sutton S. Randomized controlled trial evaluation of a tailored leaflet and SMS text message self-help intervention for pregnant smokers (MiQuit). Nicotine Tob Res. 2012 May;14(5):569-77. PMID: 22311960. doi: ntr254.[pii] 10.1093/ntr/ntr254.

27. Naughton F, Jamison J, Sutton S. Attitudes towards SMS text message smoking cessation support: a qualitative study of pregnant smokers. Health Educ Res. 2013 Oct;28(5):911-22. PMID: 23640985. doi: cyt057.[pii]10.1093/her/cyt057.

28. Pollak KI, Lyna P, Bilheimer A, Farrell D, Gao X, Swamy GK, et al. A pilot study testing SMS text delivered scheduled gradual reduction to pregnant smokers. Nicotine Tob Res. 2013 Oct;15(10):1773-6. PMID: 23569007. doi: ntt045.[pii] 10.1093/ntr/ntt045.

29. Davis AM, Wambach KA, Nelson EL, Odar C, Lillis T, McKinley A, et al. Health behavior change in pregnant women: a two-phase study. Telemed J E Health. 2014 Dec;20(12):1165-9. PMID: 25289706. doi: 10.1089/tmj.2013.0374.

30. Abroms LC, Johnson PR, Heminger CL, Van Alstyne JM, Leavitt LE, Schindler-Ruwisch JM, et al. Quit4baby: results from a pilot test of a mobile smoking cessation program for pregnant women. JMIR Mhealth Uhealth. 2015 Jan 23;3(1):e10. PMID: 25650765. doi: v3i1e10.[pii] 10.2196/mhealth.3846.
