# Supplementary material for: The Usability and Effectiveness of Mobile Health Technology–Based Lifestyle and Medical Intervention Apps Supporting Health Care During Pregnancy: Systematic Review
Source: JMIR Mhealth Uhealth. 2018 Apr 24;6(4):e109. doi: 10.2196/mhealth.8834 (PMC5941088; doi:10.2196/mhealth.8834)
Supplement: Multimedia Appendix 7 [file mhealth_v6i4e109_app7.pdf]

Multimedia Appendix 7. Overview of studies reporting on lifestyle mHealth apps: acceptability.

**Author**<br><br>**Year**	**Technique**	**Focus**	**Acceptability**		
			**User satisfaction**	**Suitability**	**Recommendation**
van Zutphen et al<br><br>2008 [17]	Email containing a quiz with a maximum of six questions, tailored to the stage of the pregnancy on health related topics (nutrition, exercise, lifestyle, smoking, safety)	Fruit, vegetables, smoking, folic acid, alcohol		81% of respondents (n=163) evaluated the program as reliable.	
Bot et al<br><br>2009 [18]	Emails containing quizzes on the topics: nutrition, smoking, physical activity, safety, lifestyle or care and pregnancy every 4 weeks	Fruit,<br><br>vegetables, smoking, folic acid, alcohol		78% of the respondents (n=3763) showed a high or intermediate appreciation.	
Dalrymple et al<br><br>2013 [19]	Text messaging three times a week until a baby is 1 year of age; based on topics discussed in prenatal classes	Fruit, vegetables, smoking, folic acid, alcohol	All respondents found the program was "very" or "somewhat" helpful. (n=7) .	All respondents found the program was "very" or "somewhat" interesting. (n=7)	
Pollak et al<br><br>2014 [23]	SMS- texting intervention (PregCHAT) vs a generic texting intervention TexT4baby):PregCHAT = Personalized feedback based on women's intake of sweetened beverages, fruits and vegetables, fast food, daily steps	Weight control	Women in the PregCHAT text arm rated the program as helpful (64% rated 5 or higher on a 7-point Likert		80 % of the women in the PregCHAT text arm would recommend the program to a friend.

	taken and weight<br><br>TexT4baby = general information to improve health		item)		
Knight-Agarwall et al<br><br>2015 [24]	Eating4Two app which provided a graphing function to record the weight through the pregnancy with real-time feedback on weight gain and general information on antenatal nutrition	Weight control	70% found the weight gain graph helpful. 80% found the nutrition advise helpful or very helpful.	Participants enjoyed using the application.	
Soltani et al<br><br>2015 [25]	MOMTech: Text messages twice a day including self-monitoring weight management and feedback on setting goals	Weight control		Participants liked the holistic nature of the intervention	
Herring et al<br><br>2016 [31]	Skills training and self-monitoring texts with personalized feedback by daily messages tailored to each behavioral goal to build skills and self-efficacy	Weight control	96% reported that the skills they learned in the program were extremely helpful (at least an 8 on a 10-point scale); 96% found the text messages extremely useful. 82% found the coach calls extremely		

			useful. 87% reported the program was extremely successful in changing eating habits		
Naughton et al 2012 [26]	Tailored self-help leaflet followed by an 11-week program of tailored text messages or (controls) a nontailored self-help leaflet	Smoking cessation	Between 81% and 96% of the MiQuit participants rated the messages as helpful to some degree.		
Pollak et al<br><br>2013 [28]	SMS-delivered support messages or support messages plus a scheduled gradual reduction (SGR)	Smoking cessation	Women found the program helpful (mean score of 6 out of 7 in a 7-point scale).	Women found the program to be acceptable (mean score of 6 out of 7 in a 7-point scale)	Most women would recommend the program to a friend, with 78% in both arms reporting 7 in a 7-point scale.
Davis et al<br><br>2014 [29]	Daily text messages and three 20-min voice-over-Powerpoint weekly	Smoking cessation mental Health	All participants found the text messages useful (n=5).		
Abroms et al<br><br>2015 [30]	Either receiving sms-text messaging for the TexT4baby program to improve health or receiving sms-text messaging for the TexT4baby program as well as Quit4Baby	Smoking cessation	All respondents (n=16): "agreed" and "completely agreed" that the	Most participants liked the program overall.	All respondents (n=16) would recommend the program to a friend.

	program which aimed to improve self-efficacy for smoking cessation		program was helpful in quitting.		

References

17. van Zutphen M, Milder IE, Bemelmans WJ. Usage of an online healthy lifestyle program by pregnant women attending midwifery practices in Amsterdam. Prev Med. 2008 Jun;46(6):552-7. PMID: 18302971. doi: S0091-7435(08)00006-6.[pii] 10.1016/j.ypmed.2008.01.003.

18. Bot M, Milder IE, Bemelmans WJ. Nationwide implementation of Hello World: a Dutch email-based health promotion program for pregnant women. J Med Internet Res. 2009 Jul 30;11(3):e24. PMID: 19674957. doi: v11i3e24.[pii] 10.2196/jmir.1183.

19. Dalrymple PW, Rogers M, Zach L, Turner K, Green M. Collaborating to develop and test an enhanced text messaging system to encourage health information seeking. J Med Libr Assoc. 2013 Jul;101(3):224-7. PMID: 23930095. doi: 10.3163/1536-5050.101.3.014 JMLA-D-12-00077.[pii]

23. Pollak KI, Alexander SC, Bennett G, Lyna P, Coffman CJ, Bilheimer A, et al. Weight-related SMS texts promoting appropriate pregnancy weight gain: a pilot study. Patient Educ Couns. 2014 Nov;97(2):256-60. PMID: 25153313. doi: S0738-3991(14)00304-8.[pii] 10.1016/j.pec.2014.07.030.

24. Knight-Agarwal C, Davis DL, Williams L, Davey R, Cox R, Clarke A. Development and Pilot Testing of the Eating4two Mobile Phone App to Monitor Gestational Weight Gain. JMIR Mhealth Uhealth. 2015 Jun 05;3(2):e44. PMID: 26048313. doi: v3i2e44.[pii] 10.2196/mhealth.4071.

25. Soltani H, Duxbury AM, Arden MA, Dearden A, Furness PJ, Garland C. Maternal Obesity Management Using Mobile Technology: A Feasibility Study to Evaluate a Text Messaging Based Complex Intervention during Pregnancy. J Obes. 2015;2015:814830. PMID: 25960889. doi: 10.1155/2015/814830.

26. Naughton F, Prevost AT, Gilbert H, Sutton S. Randomized controlled trial evaluation of a tailored leaflet and SMS text message self-help intervention for pregnant smokers (MiQuit). Nicotine Tob Res. 2012 May;14(5):569-77. PMID: 22311960. doi: ntr254.[pii] 10.1093/ntr/ntr254.

28. Pollak KI, Lyna P, Bilheimer A, Farrell D, Gao X, Swamy GK, et al. A pilot study testing SMS text delivered scheduled gradual reduction to pregnant smokers. Nicotine Tob Res. 2013 Oct;15(10):1773-6. PMID: 23569007. doi: ntt045.[pii] 10.1093/ntr/ntt045.

29. Davis AM, Wambach KA, Nelson EL, Odar C, Lillis T, McKinley A, et al. Health behavior change in pregnant women: a two-phase study. Telemed J E Health. 2014 Dec;20(12):1165-9. PMID: 25289706. doi: 10.1089/tmj.2013.0374.

30. Abroms LC, Johnson PR, Heminger CL, Van Alstyne JM, Leavitt LE, Schindler-Ruwisch JM, et al. Quit4baby: results from a pilot test of a mobile smoking cessation program for pregnant women. JMIR Mhealth Uhealth. 2015 Jan 23;3(1):e10. PMID: 25650765. doi: v3i1e10.[pii] 10.2196/mhealth.3846.

31. Herring SJ, Cruice JF, Bennett GG, Rose MZ, Davey A, Foster GD. Preventing excessive gestational weight gain among African American women: A randomized clinical trial. Obesity (Silver Spring). 2016 Jan;24(1):30-6. PMID: 26592857. doi: 10.1002/oby.21240
