# Supplementary material for: The Usability and Effectiveness of Mobile Health Technology–Based Lifestyle and Medical Intervention Apps Supporting Health Care During Pregnancy: Systematic Review
Source: JMIR Mhealth Uhealth. 2018 Apr 24;6(4):e109. doi: 10.2196/mhealth.8834 (PMC5941088; doi:10.2196/mhealth.8834)
Supplement: Multimedia Appendix 8 [file mhealth_v6i4e109_app8.pdf]

Multimedia Appendix 8. Overview of studies reporting on lifestyle mHealth apps: effectiveness.

Author and year	Technique	Focus	Effectiveness
van Dijk et al 2016 [35]	Personal Web-based coaching based on identified nutrition and lifestyle risks factors at baseline by email and text messaging with a maximum of 3 per week	Fruit, vegetables, smoking, folic acid, and alcohol	Effectiveness on all patients: vegetable intake: improvement of 20.9% (6 weeks) and increase up to 26.3% (6 months). Fruit intake improvement by 36.1% and 38.4% at 6 weeks and 6 months, inadequate folic acid use: decrease of 53.6% and 56.3%, tobacco and alcohol use were reduced by 23.8% and 27.0% at 6 weeks and 6 months
Evans et al 2015 [34]	Text messages 3 times a week, which were tailored to the gestational age focusing on intake of fruit, vegetables, alcohol, and prenatal vitamins as well as on smoking	Fruit, vegetables, smoking, alcohol, and prenatal vitamins	No significant difference in health behavior between intervention and controls. Significant lower self-reported alcohol consumption in high-dosage intervention group
Pollak et al 2014 [23]	SMS[a]-texting intervention (PregCHAT) vs a generic texting intervention TexT4baby): PregCHAT=Personalized feedback based on women's intake of sweetened beverages, fruits and vegetables, fast food, daily steps taken, and weight<br><br>TexT4baby=general information to improve health	Weight control	There was a nonsignificant difference in mean gestational weight gain 6 pounds less for women who completed the intervention, between the PregCHAT arm and the Txt4Baby arm. No differential treatment effect was found in self-reported physical activity or the nutrition score
Soltani et al 2015 [25]	MOMTech: Text messages twice a day including self-monitoring weight management and feedback on setting goals	Weight control	Mean gestational weight gain; 6.65 in intervention group vs 9.74 in controls. (Note well: No statistical analysis were performed due to small sample size)
Herring et al 2016 [31]	Skills training and self-monitoring texts with personalized feedback by daily messages tailored to each behavioral goal	Weight control	Weight gain was significantly reduced in the intervention group compared with usual care (37% vs 66%, *P*=.03). No significant differences were found in the neonatal or obstetric outcomes

	to build skills and self-efficacy		
Choi et al<br>2015 [22]	SMS-texting intervention; daily messages and a mobile phone activity diary with automated feedback and self-monitoring systems	Physical activity	Intervention participants tend to increase in daily steps compared with control participants, but not significantly different
Fuijoka et al<br>2012 [32]	e-learning program to support pregnant women who want to quit smoking	Smoking cessation	The rate of nonsmoking was 71.1% of participants who completed the study (n=48). Carbon monoxide exhalation levels significantly decreased (from 6.43±4.5 ppm at the beginning to 0.7±1.0 ppm in 1 month, to 0.29±1.08 in 3 months (*P*<.001).
Naughton et al<br>2012 [26]	Tailored self-help leaflet followed by an 11-week program of tailored text messages or (controls) a nontailored self-help leaflet	Smoking cessation	At 3-month follow-up, there was no statistically significant difference between intervention and controls. (NB: the trial was not powered to detect a group difference on smoking outcomes)
Pollak et al<br>2013 [28]	SMS-delivered support messages or support messages plus a scheduled gradual reduction (SGR)	Smoking cessation	The intervention group tends to a lower prevalence of smoking: 7-day point prevalence of 13.4% vs 7.5% in favor of the SGR arm. (NB: given the small sample size, the purpose of the arms is simply to monitor for unexpected, gross differences between arms)
Moniz et al<br>2015 [33]	12 weekly received text messages encouraging preventive health behaviors (tobacco cessation, condom use for disease prevention, nutrition optimization, seat belt use, and breastfeeding)	Smoking cessation, nutrition	Improvements in self-reported behavior between baseline and follow-up: decreased tobacco use in 41% of the smokers (n=44). More consistent condom use in 7% of the sexually active participants (n=83). Moreover, 32% of the participants reported more prenatal vitamin intake; 32% of the participants reported more frequent seatbelt use; and 41% of the participants reported more frequent healthy food intake

[a]SMS: short message service.

References

22. Choi J, Lee JH, Vittinghoff E, Fukuoka Y. mHealth Physical Activity Intervention: A Randomized Pilot Study in Physically Inactive Pregnant Women. Matern Child Health J. 2016 May;20(5):1091-101. PMID: 26649879. doi: 10.1007/s10995-015-1895-7 10.1007/s10995-015-1895-7.[pii]

23. Pollak KI, Alexander SC, Bennett G, Lyna P, Coffman CJ, Bilheimer A, et al. Weight-related SMS texts promoting appropriate pregnancy weight gain: a pilot study. Patient Educ Couns. 2014 Nov;97(2):256-60. PMID: 25153313. doi: S0738-3991(14)00304-8.[pii] 10.1016/j.pec.2014.07.030.

25. Soltani H, Duxbury AM, Arden MA, Dearden A, Furness PJ, Garland C. Maternal Obesity Management Using Mobile Technology: A Feasibility Study to Evaluate a Text Messaging Based Complex Intervention during Pregnancy. J Obes. 2015;2015:814830. PMID: 25960889. doi: 10.1155/2015/814830.

26. Naughton F, Prevost AT, Gilbert H, Sutton S. Randomized controlled trial evaluation of a tailored leaflet and SMS text message self-help intervention for pregnant smokers (MiQuit). Nicotine Tob Res. 2012 May;14(5):569-77. PMID: 22311960. doi: ntr254.[pii] 10.1093/ntr/ntr254.

28. Pollak KI, Lyna P, Bilheimer A, Farrell D, Gao X, Swamy GK, et al. A pilot study testing SMS text delivered scheduled gradual reduction to pregnant smokers. Nicotine Tob Res. 2013 Oct;15(10):1773-6. PMID: 23569007. doi: ntt045.[pii] 10.1093/ntr/ntt045.

31. Herring SJ, Cruice JF, Bennett GG, Rose MZ, Davey A, Foster GD. Preventing excessive gestational weight gain among African American women: A randomized clinical trial. Obesity (Silver Spring). 2016 Jan;24(1):30-6. PMID: 26592857. doi: 10.1002/oby.21240

32. Fujioka N, Kobayashi T, Turale S. Short-term behavioral changes in pregnant women after a quit-smoking program via e-learning: a descriptive study from Japan. Nurs Health Sci. 2012 Sep;14(3):304-11. PMID: 22950611. doi: 10.1111/j.1442-2018.2012.00702.x.

33. Moniz MH, Meyn LA, Beigi RH. Text Messaging to Improve Preventive Health Attitudes and Behaviors During Pregnancy: A Prospective Cohort Analysis. J Reprod Med. 2015 Sep-Oct;60(9-10):378-82. PMID: 26592061.

34. Evans W, Nielsen PE, Szekely DR, Bihm JW, Murray EA, Snider J, et al. Dose-response effects of the text4baby mobile health program: randomized controlled trial. JMIR Mhealth Uhealth. 2015 Jan 28;3(1):e12. PMID: 25630361. doi: v3i1e12.[pii] 10.2196/mhealth.3909.

35. van Dijk M, Huijgen NA, Willemsen SP, Laven JSE, Steegers EAP, Steegers-Theunissen RPM. Impact of an mHealth platform for pregnancy on nutrition and lifestyle of the reproductive population: a survey. JMIR mHealth uHealth, 2016 apr-jun; 4 (2): e53.
