# Supplementary material for: The Usability and Effectiveness of Mobile Health Technology–Based Lifestyle and Medical Intervention Apps Supporting Health Care During Pregnancy: Systematic Review
Source: JMIR Mhealth Uhealth. 2018 Apr 24;6(4):e109. doi: 10.2196/mhealth.8834 (PMC5941088; doi:10.2196/mhealth.8834)
Supplement: Multimedia Appendix 9 [file mhealth_v6i4e109_app9.pdf]

Appendix 6. Complete overview of results on mHealth medical applications

Author year	Focus	Usability				Acceptability			Effectiveness
		**Actual use**	**Interest**	**Appropriateness**	**Ability**	**User satisfaction**	**Appreciation**	**Recommendation**	
Zairina 2015	Asthma								The changes in ACQ score from baseline to 3 months for MASTERY and usual care groups were -0.01 ± 0.11 and 0.16± 0.09. No significant difference in lung function.
Carral 2015	Diabetes								No significant difference in HbA1c levels. Significantly less insulin treatment and less health care visits in intervention group
Homko 2012	Diabetes								No significant differences between the two groups in regard to maternal blood glucose values or infant birth weight.
Homko 2007	Diabetes								No significant difference between the two groups blood glucose values and HbA1c levels. Significantly more women in the Internet group received insulin therapy (31% vs. 4%; P <0.05). There were no significant differences in pregnancy and neonatal outcomes between the two groups.
Hirst 2015	Diabetes					90% of the participants agreed or strongly agreed the management system is convenient.	83% of the participants agreed or strongly agreed the management system is reliable.		
Nicholson 2016	Diabetes	65% of the participants logged in to the website at least 3 times during pregnancy		most participants (*n* = 8) thought the website was user-friendly and easy to access	Women reported little to no experience with online discussion groups, but expressed a		Average gestational weight gain for all participants was 19.9lbs ± 13.2. No statistically significant		

		"Using this program would probably…would be the first for me because I don't do the message boards and things of that nature, but I'm willing to give it a try, just, you know, because somebody may know something more than I do, and it never hurts to ask."			willingness to use a message board to communicate with other women with GDM		difference between baseline and 36 weeks of gestation in HbA1c levels.		
Perez-Ferre 2010	Diabetes								No difference in maternal metabolic parameters nor in pregnancy outcome.
Stockwell 2014	Influenza vaccination								Women in the intervention group were more likely to receive an influenza vaccination (AOR 1.3, CI 1.003-1.69).
Jordan 2015	Influenza vaccination								No significant increase of the odds of vaccination at follow-up. Significant increase of continued intent to be vaccinated later in the season (AOR 2.1, 95% CI 1.4, 3.1)
Yudin 2017	Influenza vaccination								No significant difference between the intervention and control group.
